# Induction of labor and early-onset Sepsis guidelines: impact on NICU admissions in Erie County, NY

**DOI:** 10.1186/s40748-019-0114-8

**Published:** 2019-12-05

**Authors:** Vikramaditya Dumpa, Indira Avulakunta, James Shelton, Taechin Yu, Satyan Lakshminrusimha

**Affiliations:** 1 Division of Neonatology, Department of Pediatrics, NYU Winthrop Hospital, 259 First St, Mineola, New York, 11501 USA; 2 Department of Pediatrics, Brookdale University Hospital and Medical, 1 Brookdale Plaza, Brooklyn, New York, 11212 USA; 3 Department of Obstetrics and Gynecology, Oishei Children’s Hospital, 1001 Main St, Buffalo, New York, 14203 USA; 4 Department of Obstetrics and Gynecology, Holy Redeemer Health System, 667 Old Welsh Rd, Huntingdon Valley, PA 19006 USA; 5 Department of Pediatrics, UC Davis Children’s Hospital, 2516 Stockton Blvd, Sacramento, California, 95817 USA

**Keywords:** Early-term, ACOG guidelines, Induction of labor, Early-onset sepsis

## Abstract

**Background:**

Elective delivery prior to term gestation is associated with adverse neonatal outcomes. The impact of American College of Obstetricians and Gynecologists (ACOG) guidelines recommending against induction of labor (IOL) < 39 weeks’ postmenstrual age (PMA) on the frequency of early-term births and NICU admissions in Erie County, NY was evaluated in this study.

**Methods:**

This is a population-based retrospective comparison of all live births and NICU admissions in Erie County, NY between pre-and post-ACOG IOL guideline epochs (2005–2008 vs. 2011–2014). Information on early-term, full/late/post-term births and NICU admissions was obtained. A detailed chart analysis of indications for admission to the Regional Perinatal Center was performed.

**Results:**

During the 2005–2008 epoch, early-term births constituted 27% (11,968/44,617) of live births. The NICU admission rate was higher for early-term births (1134/11968 = 9.5%) compared to full/late/post-term (1493/27541 = 5.4%).

In the 2011–2014 epoch, early-term births decreased to 23% (10,286/44,575) of live births. However, NICU admissions for early-term (1072/10286 = 10.4%) and full/late/post-term births (1892/29508 = 6.4%) did not decrease partly due to asymptomatic infants exposed to maternal chorioamnionitis admitted for empiric antibiotic therapy as per revised early-onset sepsis guidelines.

**Conclusions:**

ACOG recommendations against elective IOL or cesarean delivery < 39 weeks PMA were rapidly translated to clinical practice and decreased early-term births in Erie County, NY. This decrease did not translate to reduced NICU admissions partly due to increased NICU admissions for empiric antibiotic therapy.

## Background

Research has convincingly demonstrated that neonatal outcomes improve with increasing gestational age, with the best outcomes seen in infants delivered at 39–40 weeks post-menstrual age (PMA) [1, 2]. The American College of Obstetricians and Gynecologists (ACOG) has restricted elective term delivery to women with a confirmed gestational age of at least 39 weeks for the past two decades. In spite of these recommendations, elective deliveries at less than 39 weeks PMA were not uncommon [3]. When the increased neonatal morbidity in infants delivered electively prior to 39 weeks became a public health priority, ACOG published new guidelines in 2009 strongly recommending against elective induction before 39 weeks [4]. These guidelines have reduced early-term deliveries without reducing neonatal intensive care admissions [5].

Our current study focuses on the impact of the 2009 ACOG guidelines on early-term births and neonatal intensive care unit (NICU) admissions in Erie County, NY. We have previously shown that early-term births are associated with an increased risk of NICU admission in our County (8.8% vs. 5.3% in full/late term gestation) [6]. We hypothesized that ACOG’s induction of labor (IOL) guidelines would result in a decrease in the early-term births which in turn would decrease NICU admissions. We also investigated the effect of revised early-onset sepsis management guidelines from 2010 on NICU admissions [7, 8].

## Methods

The study was approved by the institutional review board of The State University of New York at Buffalo, NY. This is a population based retrospective study consisting of all live births in Erie County, New York from January 1, 2005 to December 31, 2008 and January 1, 2011 to December 31, 2014. For this study, we used the following definitions of birth based on gestational age: (i) early-term birth (born between 37 ^0/7^ and 38 ^6/7^ weeks’ PMA), (ii) full-term birth (born between 39 ^0/7^ and 40 ^6/7^ weeks’ PMA), late-term birth (born between 41 ^0/7^ and 41 ^6/7^ weeks’ PMA) and post-term birth (born at or after 42 ^0/7^ weeks). These definitions are in accordance with the ‘Term’ pregnancy workgroup recommendations [9]. Erie County has four birthing hospitals, comprising one regional perinatal center (RPC), one level III, and two level II NICUs.

Maternal and Neonatal Characteristics: The study period was divided into 2 epochs, pre-ACOG guideline epoch (2005–2008) and post-ACOG guideline epoch (2011–2014). Data from 2009 (the year of release of the guidelines) and 2010 were excluded as wash-out period. The number of term births and NICU admissions in Erie County was studied during these time periods. Preterm births and preterm NICU admissions were excluded from further analysis in the study. The impact of the guidelines was observed starting from 2011 taking into account a presumed one-year lag time between recommendations and their translation and adoption to clinical practice.

In order to explain trends in admissions to NICU, we performed further analysis of all NICU admissions to the RPC at Women and Children’s Hospital of Buffalo during both epochs. Pertinent baseline maternal and neonatal characteristics including admission indications to the NICU and length of stay were extracted from individual medical records. The RPC accounts for up to half of the NICU admissions in Erie County. Since the neonatal management protocols are the same in all the four NICUs, we feel that the findings noted reflect the patterns in the County.

Admission to the NICU or neonatologist’s service was considered as the primary outcome measure to determine neonatal morbidity.

The following definitions were used for individual patient chart analysis:

(i) Chorioamnionitis was clinically diagnosed with presence of maternal fever (intrapartum temperature ≥ 38 °C), and uterine tenderness or foul-smelling amniotic fluid.
(ii) Symptomatic neonate: signs and symptoms in the term newborn infant including any form of respiratory distress, cardiovascular instability, feeding difficulties, hypoglycemia or abnormal temperature.
(iii) Abnormal labs in the neonate were defined as immature bands to total segmented neutrophil (IT) ratio of ≥0.3, neutropenia with absolute neutrophil count (ANC) less than 1000 cells/μL, leukopenia with white blood cell (WBC) count less than 5000 cells/μL, leukocytosis with WBC count greater than 30,000 cells/μL, or C-reactive protein (CRP) > 10 mg/L. IT ratio of ≥0.3 is considered abnormal based on the normative reference range at the institution’s laboratory.

### Statistical analysis

Qualitative variables were expressed as percentages, and bivariate analyses were performed using the χ2 test. All analyses were performed using statistical software (Graphpad Prism, Graphpad software, La Jolla CA). *P* value ≤0.05 was considered statistically significant.

Sample size Calculation: We planned a study comparing NICU admissions among early, full and late-term births with an equal number of births in a pre-guideline epoch and post-guideline epoch. Prior data from Erie County NY suggested that the NICU admission rate among 37 to 41 week gestation births was 0.0664 (6.64%) [6]. If the true NICU admission rate for post-guideline change epoch decreased by 10% to 0.05976 (5.976%), we needed to study 28,178 pre-epoch births at 37–41 week gestation and 28,178 births at the same gestation in the post-epoch to be able to reject the null hypothesis that the NICU admission rates for 37–41 week gestation births before and after the IOL ACOG guideline are equal with probability (power) 0.9. The Type I error probability associated with this test of this null hypothesis is 0.05. We used an uncorrected chi-squared statistic to evaluate this null hypothesis.

By utilizing births over a period of 4 years in each epoch, we had 39,509 births at the desired gestation in the 2005–08 period. With this sample size, we were able to reject the null hypothesis that the admission rates before and after implementation of IOL guidelines are equal with probability (power) of 0.97.

## Results

In the pre-ACOG IOL guideline epoch, there were 44,617 live births over a 4-year period (2005 to 2008: Table 1). Among these births, 11,968 (26.8%) were early-term and 27,541 (61.7%) were full and late term. During this period, 5816 neonates were admitted to the NICU (13% of total live births) of which 1134 infants were born at early-term and 1493 infants at full and late term. The NICU admit rate for early-term births (1134/11,968–9.47%) was significantly higher than that for full and late term births (1493/27,541–5.4%). The overall admission rate for early, full and late term births was 6.64% (2627/39,509).

**Table 1:** Total births and NICU admissions by gestational age categories during the pre-induction of labor guidelines epoch (2005–2008) and the post-guideline epoch (2011–2014)

Erie County data	2005–2008	2011–2014	*P* value
Total births	44,617	44,575	
Early term births	11,968 (26.8%)	10,286 (23.07%)	< 0.0001
Full & late term births	27,541 (61.7%)	29,508 (66.19%)	< 0.0001
Total NICU admissions	5816 (13%)	6129 (13.74%)	0.0064
Early term admissions (% of all ET births)	1134 (9.47%)	1072 (10.42%)	0.035
Full & Late term admissions (% of all FLT births)	1493 (5.4%)	1892 (6.41%)	< 0.0001
Early + Full & Late term admissions (% of ET + FLT births**)**	2627/39509 (6.64%)	2964/39794 (7.44%)	< 0.0001

ET- Early-term; FLT- Full & Late term

**Fig. 1:**
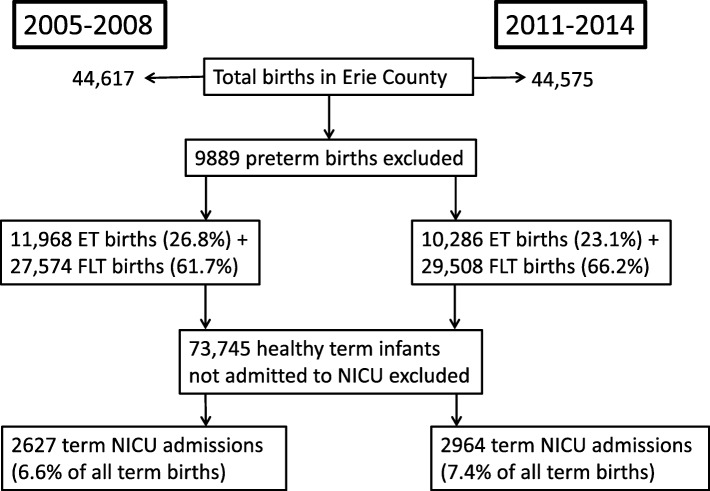
Flow chart detailing the cohort selection and comparing the term births and NICU admissions between 2005-2008 and 2011-2014. ET- Early term, FLT- Full/Late term

In the post-ACOG guideline epoch, there were 44,575 live births over a 4-year period (2011 to 2014, Table 1). A significant decrease in early-term births (10,286–23.1%) was associated with an increase in full and late term births (29,508–66.2%). During this period, 6129 (13.7% of total live births) were admitted to the NICU of which 1072 infants were born early-term and 1892 infants at full and late term gestation. 10.4% of early-term births and 6.4% of full and late term births resulted in NICU admissions. Thus, compared to the pre ACOG IOL guideline period, there was a significant increase in the number of admissions in post ACOG guideline period in both early-term and full and late term group. Combined together, NICU admissions in the post-ACOG IOL guideline epoch increased by 0.8% of all term births (from 6.64 to 7.44%, Table 1). Fig. 1 depicts the study flow diagram.

This increase in admissions in the post-ACOG period is contrary to what was expected based on a significant decrease in early-term births. We further investigated the reason for the increased term NICU admissions. We noted that there was a change in our admission practice based on the revised Center for Disease Control and Prevention (CDC) Guidelines in 2010 on Prevention of Perinatal Group B Streptococcal disease and a subsequent Clinical Report from the American Academy of Pediatrics (AAP) on approach to infants with maternal chorioamnionitis. The 2010 CDC guidelines stated that ‘In an effort to avert neonatal infections, maternal fever alone in labor may be used as a sign of chorioamnionitis and hence indication for antibiotic treatment, particularly among women with a significant risk factor for chorioamnionitis (e.g., prolonged labor or prolonged rupture of membranes).’ [7] The AAP policy on recommendation for the prevention of early-onset sepsis in newborns from 2011 stated that ‘All well-appearing newborn infants born to women given a diagnosis of chorioamnionitis by their obstetrical provider should undergo a limited diagnostic evaluation (no lumbar puncture) and receive empirical antimicrobial therapy.’ [8]

We further analyzed the data from the RPC to investigate the cause of increased admissions to the NICU and indications for admission in the 2011–14 epoch. We observed a marked increase in the number of asymptomatic newborn infants born to mothers with chorioamnionitis in the post-IOL guidelines. These infants were admitted for empiric antibiotic therapy. Infants requiring intravenous antibiotic therapy are transferred to the NICU in all the birthing hospitals in Erie County, NY. Admissions to the NICU with a primary indication of maternal chorioamnionitis increased from 0.25% (26/10158) to 1.9% (190/9948) of all live births at the RPC. Of the NICU admissions secondary to maternal chorioamnionitis, 31% of neonates were asymptomatic during 2005–2008 compared to 72% in 2011–2014.

**Fig. 2:**
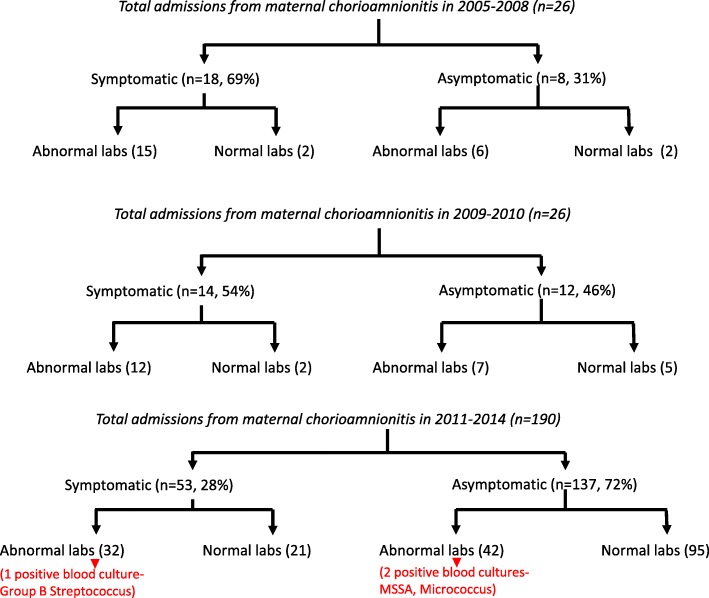
Flow charts depicting the number of admissions resulting from maternal chorioamnionitis in the study period, divided into symptomatic and asymptomatic infants with normal/abnormal labs

One symptomatic infant (presented with fever, tachypnea, and hypoxemia) and two asymptomatic infants during the entire study period had positive blood cultures as shown in Fig. 2. The two asymptomatic infants admitted exclusively due to maternal chorioamnionitis had abnormal labs with bandemia. The blood cultures were positive for methicillin sensitive *Staphylococcus aureus* and Micrococcus respectively and were treated with a full course of antibiotics.

## Discussion

Changes in health-care policies have a significant impact on healthcare utilization and cost. Obstetric practices such as cesarean delivery and IOL prior to 39 weeks PMA are associated with increased neonatal morbidity necessitating admission to the NICU.

The increase in cesarean delivery and induction rates led to an increase in the number of preterm births or early-term births. Research has shown that the neonatal outcomes improve with advancing gestational age with better outcomes seen in infants delivered around 39 weeks. The implications of delivery at early-term gestational age seem to extend beyond the neonatal period, with associated increased mortality observed in infancy, early childhood and young adulthood among infants born at early-term [3, 10–17].

All these findings have stimulated a national effort to reduce elective delivery before 39 weeks through increased adherence to the ACOG’s recommendations. In addition, quality organizations such as Joint Commission, National Quality Forum and Institute for Healthcare Improvement have focused on prevention of elective deliveries prior to 39 weeks as a measure of quality [18]. Quality improvement projects launched recently aiming at reducing the number of elective early-term births have been successful [19, 20]. Several initiatives from the New York State Department of Health (NYSDOH) such as the State Perinatal Quality Collaborative, Obstetrical Prenatal Education Project and implementation of Community Health Collaborative by the Buffalo prenatal-perinatal network may have contributed to a reduction in early-term deliveries. In addition, analyses of the data from the U. S Vital statistics also report a decline in early-term deliveries gradually from 2005 to 2012 [21].

Studies have shown decreased NICU admissions after implementation of local hospital policies against elective deliveries before 39 completed weeks of gestation. In a retrospective study involving more than 24,000 births comparing the neonatal admissions before and after implementation of guidelines restricting elective deliveries to 39 weeks and above, a statistically significant decrease was noted in the number of admissions [22]. Similarly, Oregon’s hard-stop policy limiting elective early-term deliveries reduced the number of babies delivered at less than 39 weeks PMA [5]. Similar to our study, these investigators did not observe a reduction in NICU admissions.

To our knowledge, there is no study comparing neonatal morbidity and indications for neonatal NICU admission across the term gestation specifically before and after implementation of the 2009 ACOG guidelines. We had hypothesized that neonatal morbidity and admission rate to the NICU would decrease after implementation of the ACOG guidelines. Nevertheless, as evident from our results, even though there was a decrease in the early-term deliveries, there was an overall increase in the NICU admissions in the post ACOG implementation period. A similar trend was observed by Parikh et al. with a reduction in elective inductions prior to 39 weeks PMA from 2008 to 2011 countered by an increase in NICU utilization. The authors comment that this unexpected observation may be due to specific NICU practices at each institution during the years examined [23].

The observed increase in NICU admissions in our study can be partly attributed to changes in practice. In 2010, the CDC in collaboration with the American Academy of Family Physicians, AAP, American College of Nurse-Midwives, ACOG, and American Society for Microbiology published revised group B streptococcal (GBS) guidelines entitled “Prevention of perinatal group B streptococcal disease: revised guidelines from CDC 2010” in the Morbidity and Mortality Weekly Report [7]. In 2011, the Committee on Infectious Diseases and the Committee on Fetus and Newborn (COFN) published a Clinical Report in Pediatrics that was in agreement with the 2010 GBS guidelines and included the same algorithm. This statement recommended that all well appearing newborn infants born to women given a diagnosis of chorioamnionitis by their obstetrical provider should undergo a limited diagnostic evaluation and receive empirical antimicrobial therapy [8]. Antibiotics could be discontinued after 48 h if the blood culture did not show bacterial growth, the infant remained asymptomatic, and laboratory study findings are normal. However, if laboratory test results were abnormal and the mother received intrapartum antibiotics, COFN did not provide guidelines for the duration of antibiotics in an asymptomatic infant with sterile blood culture in this report [24, 25].

There was a significant increase in the number of NICU admissions from maternal chorioamnionitis after the policy change with the majority of infants being asymptomatic (Fig. 2). More than half of the increase in the admissions could be attributed to the admission of asymptomatic infants with maternal chorioamnionitis. However, there were two positive blood cultures (Methicillin sensitive *Staphylococcus aureus* and Micrococcus) with abnormal CBC findings during the study period among the infants admitted with maternal chorioamnionitis, with a rate of 0.9%. While the possibility of contamination cannot be ruled out, the presence of leukocytosis and a significant left-shift on CBC along with a positive blood culture led to cerebrospinal fluid analysis and a complete course of treatment with antibiotics.

There is considerable debate on the utility of the above guidelines especially concerning the treatment of asymptomatic newborns with a risk factor of maternal chorioamnionitis [26–29]. In studies looking at early-onset sepsis in late-preterm and term infants exposed to maternal chorioamnionitis, the rates of positive blood culture at ≤72 h are very low, ranging from 0 to 1.24%, which is similar to that observed in our study [26, 30–32]. In light of all these observations, it was later suggested that well-appearing late-preterm and term infants should be managed with close clinical observation, because of the low sensitivity of risk factors in the ascertainment of early-onset sepsis in this group [33]. The most recent AAP clinical report recommends a risk stratification approach in the management of late preterm and term neonates with suspected early-onset sepsis [34]. Three options are suggested – (a) use of categorical algorithms in which threshold values for intrapartum risk factors are used; (b) multivariate risk assessment model based on both intrapartum risk factors and physical examination (such as the Neonatal Early-Onset Sepsis Risk Calculator); and (c) serial physical examinations to detect presence of clinical signs of illness after birth. The increased NICU admissions observed in our study would not have occurred with the last two options outlined in the new AAP algorithm.

The clinical-sign based approach (third option) suggested by the recent AAP clinical report would have missed the two culture-positive infants in our study who were completely asymptomatic and were evaluated and received early antibiotics exclusively because of maternal chorioamnionitis. In support of the revised AAP Clinical Report, the frequency of positive blood culture in the presence of maternal chorioamnionitis and abnormal neonatal laboratory findings was low (3/74 infants). Positive blood culture results were not different among symptomatic and asymptomatic infants in our cohort (1/32 vs. 2/42 respectively – Fig. 2).

Our study has several limitations. The detailed maternal indications for early-term deliveries were not evaluated. This would preclude us from knowing the percentage of medically indicated deliveries, spontaneous deliveries, and elective deliveries. The determination of gestational age is based on the first trimester ultrasonography in most of the neonates. However, the accuracy of the gestational age in the rest of the neonates was based on the best obstetric and/or neonatal estimate and may not be accurate. The indications for admission to NICU were studied at the RPC only. Also, since all the infants are admitted to neonatologist’s service or NICU for intravenous antibiotics in Erie County, these data may not be reflective of practices where infants are managed in normal newborn nursery with intravenous antibiotics. Lastly, we did not study all the maternal and neonatal characteristics of infants included in the study. There could be factors other than exposure to maternal chorioamnionitis which might have led to an increase in the NICU admissions in the latter epoch.

## Conclusions

ACOG guidelines of recommendations against elective induction of labor or cesarean delivery before 39 weeks’ gestational age effectively decreased early-term births in Erie County, NY. This decrease in number of births did not translate to a reduction in NICU admissions. This is partly due to an increased number of admissions of infants with maternal chorioamnionitis. The current revision of early-onset sepsis clinical report by the AAP offering choices and not mandating empiric antibiotic therapy for asymptomatic term infants is likely to reduce NICU admissions and cost [34].

## Data Availability

The data sets used and/or analyzed during the current study are available from the corresponding author on reasonable request.

## Abbreviations

AAP: American Academy of Pediatrics
ACOG: American College of Obstetricians and Gynecologists
ANC: Absolute Neutrophil Count
CDC: Center for Disease Control and Prevention
COFN: Committee on Fetus and Newborn
CRP: C-reactive protein
GBS: Group B Streptococcal
IOL: Induction of Labor
IT: Immature bands to total segmented neutrophil
NICU: Neonatal Intensive Care Unit
NYSDOH: New York State Department of Health
PMA: Postmenstrual Age
RPC: Regional Perinatal Center
WBC: White Blood Cell
